# Adaptation to Shift Work: Physiologically Based Modeling of the Effects of Lighting and Shifts’ Start Time

**DOI:** 10.1371/journal.pone.0053379

**Published:** 2013-01-04

**Authors:** Svetlana Postnova, Peter A. Robinson, Dmitry D. Postnov

**Affiliations:** 1 School of Physics, The University of Sydney, Sydney, New South Wales, Australia; 2 Center for Integrated Research and Understanding of Sleep (CIRUS), The University of Sydney, Sydney, New South Wales, Australia; 3 Brain Dynamics Center, The University of Sydney, Sydney, New South Wales, Australia; 4 School of Physics, Saratov State University, Saratov, Russia; Georgia State University, United States of America

## Abstract

Shift work has become an integral part of our life with almost 20% of the population being involved in different shift schedules in developed countries. However, the atypical work times, especially the night shifts, are associated with reduced quality and quantity of sleep that leads to increase of sleepiness often culminating in accidents. It has been demonstrated that shift workers’ sleepiness can be improved by a proper scheduling of light exposure and optimizing shifts timing. Here, an integrated physiologically-based model of sleep-wake cycles is used to predict adaptation to shift work in different light conditions and for different shift start times for a schedule of four consecutive days of work. The integrated model combines a model of the ascending arousal system in the brain that controls the sleep-wake switch and a human circadian pacemaker model. To validate the application of the integrated model and demonstrate its utility, its dynamics are adjusted to achieve a fit to published experimental results showing adaptation of night shift workers (n = 8) in conditions of either bright or regular lighting. Further, the model is used to predict the shift workers’ adaptation to the same shift schedule, but for conditions not considered in the experiment. The model demonstrates that the intensity of shift light can be reduced fourfold from that used in the experiment and still produce good adaptation to night work. The model predicts that sleepiness of the workers during night shifts on a protocol with either bright or regular lighting can be significantly improved by starting the shift earlier in the night, e.g.; at 21∶00 instead of 00∶00. Finally, the study predicts that people of the same chronotype, i.e. with identical sleep times in normal conditions, can have drastically different responses to shift work depending on their intrinsic circadian and homeostatic parameters.

## Introduction

Shift work has become an essential part of our 24-h society. However, along with benefits of around-the-clock service, it leads to increased sleepiness of shift workers, which leads to accidents and work-related injuries. This can have detrimental consequences not only for the shift workers themselves, but also for people around them. This is especially the case in health care, transport, and public safety systems, where shift work is widespread, and lives depend on workers’ performance [1]–[5].

Increased sleepiness during the shifts is likely to be associated with misalignment of the circadian system and enforced sleep-wake schedules [5]–[6]. According to the two-process concept of Borbély [7] sleep-wake cycles are controlled by the circadian and homeostatic processes. The homeostatic process is responsible for the accumulation of sleep pressure during the time spent awake, and is hypothesized to be related to increase of somnogenic substances in the brain [8], or to synaptic plasticity [9]–[10], but the precise mechanisms are still unclear. The circadian process controls the nearly 24-hour periodicity of the sleep-wake cycles, and is largely regulated by the master circadian clock in the suprachiasmatic nucleus of the hypothalamus (SCN) [11]. The activity of the SCN is adjusted by a number of environmental inputs, with the strongest input being the light-dark cycle. Other inputs include meals, locomotion, and social interactions [12]. In a person exposed to a natural light-dark cycle, the peak of the circadian activity appears during daytime and its minimum during the night. This rhythm is also reflected in fluctuations of the core body temperature (CBT), which demonstrates minimum during the night, usually 2–3 hours before awakening, and maximum during daytime. The timing of the CBT minimum is traditionally used as a marker of the circadian phase, as it is a reasonably precise and noninvasive measure [13].

Together, the homeostatic and circadian processes contribute to the level of the total sleep drive and determine the timing of sleep-wake transitions. Shift work leads to changes in the light exposure and sleep times, thereby affecting both the homeostatic and circadian processes. Ideally, the workers’ circadian oscillators need to re-entrain in accord with the shift schedule; e.g., on the night shifts the maximum of circadian activity should appear during the night and minimum during the day, thus also allowing sufficient sleep time and recovery of the homeostatic sleep pressure. However, most of the time such re-entrainment does not happen even after many years of shift work, and the workers constantly perform in conditions of increased sleepiness and risk [3]–[5].

The problem of adaptation to shift work had been intensively studied in the last decades. It has been demonstrated that increased light intensity during the shifts and darkness during the breaks can significantly improve adaptation to shifts and decrease sleepiness. Likewise, some shift schedules are expected to be easier to adapt to than others (for reviews see [5]–[14]). However, given the large number of different protocols and conditions that are currently used in various industries, a quantitative method allowing prediction of the ease of adaptation to, and sleepiness on, different shift schedules would be highly desirable.

Several mathematical models of sleep and sleepiness have been developed and are able to successfully predict adaptation to some schedules (e.g., [15]–[19] and references therein). However, more general prediction of long-term dynamics on different shift schedules requires consideration of the interactions between the dynamic circadian and homeostatic mechanisms, as well as reciprocal interactions between the environment and sleep-wake dynamics, which are usually not implemented.

In our earlier work we introduced an integrated mathematical model of sleep-wake cycles that accounts for the above interactions and is capable to predict entrainment and sleepiness on different shift work schedules and in different light conditions [6]. The integrated model combines the two earlier models: the model of sleep-wake switch of Phillips and Robinson [20], and the model of the human circadian pacemaker of St. Hilaire et al. [21], both of which were validated based on a number of experimental studies [20]–[24]. The integrated model is based on the neurobiology of sleep and allows prediction of natural sleep-wake times, levels of sleep drive, and circadian phase depending on light input and schedules of forced wakefulness (such as shift work). Furthermore, it provides natural nonlinear dependencies between the circadian and homeostatic systems via physiologically based neuronal connections implemented in the model and dependence of light input on the states of sleep and wakefulness (see [6] and Methods for detail).

Noteworthy, the integrated model was not specifically developed to study shift work. Instead it accounts for known biological mechanisms of sleep regulation, and can thus be expected to be applicable to a multitude of sleep-related phenomena, including shift work. The integrated model was demonstrated to have good agreement with some experimental observations, such as forced desynchrony studies [24]. It also shows qualitative agreement with shift work studies [6], but was not, so far, tested for quantitative agreement. Therefore, in the present work we examine the model’s dynamics on a published experimental shift work protocol. As a representative example we have chosen the experimental night shifts protocol by Czeisler et al. (1990) [25], since this is one of the best known but at the same time easy to simulate studies. Once the parameters are adjusted to quantitatively match the experiment, we use the model to better understand the mechanisms underlying adaptation to night shifts and suggest more efficient conditions to promote entrainment and reduce sleepiness. In particular, we explore the effects of (i) different shift lighting intensities; (ii) morning commute light; and (iii) different shift start times on adaptation to shifts. We also address a question of how the model can be adjusted to simulate individual responses to shift protocols and how different model parameters affect adaptation.

## Materials and Methods

### Equations of the Integrated Model

**Figure 1:**
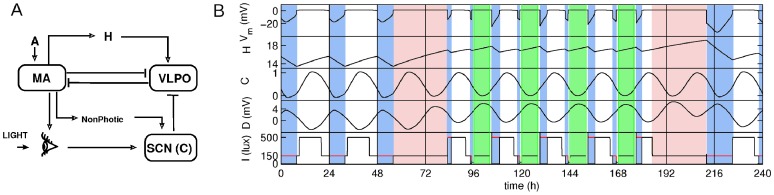
Schematic of the model and example of the dynamics. (A) – schematic of the connections among neuronal populations in the integrated model. MA refers to the wake-active monoaminergic nuclei of the brainstem and hypothalamus, VLPO to the sleep-active ventrolateral preoptic nucleus, and SCN to the suprachiasmatic nucleus of the hypothalamus. Bar-headed lines indicate inhibitory connections between neuronal populations, and arrow-headed – excitatory. (B) – Example of the dynamics of the mean MA potential *V_m_,* the homeostatic variable *H,* the circadian variable *C,* sleepiness *D*, and light input *I* in the control case. Blue-shaded areas in the plots indicate sleep intervals, green – shifts, and red – constant routines. In the bottom plot red line shows the light level as it would be present in the circadian model without the functionally important feedback from the sleep-wake switch. See text for detail.

The integrated model of the sleep-wake cycles is based on established neurobiology of sleep, and its schematic and example of dynamics are shown in Fig. 1. The essential structures for regulation of transitions between sleep and wakefulness include the wake-promoting monoaminergic (MA) group and the sleep-promoting ventrolateral preoptic nucleus (VLPO) which inhibit one another, resulting in flip-flop dynamics with only one group being active at a time: MA in wake and VLPO in sleep [26]. The state transitions are driven by inputs to this flip-flop switch, including the circadian drive from the suprachiasmatic nucleus (SCN), and the homeostatic sleep drive whose mechanisms are yet to be fully understood.

The integrated model is a combination of two earlier models: the physiologically based sleep-wake switch model of Phillips and Robinson [20], and the human circadian pacemaker model of St. Hilaire et al. [21]. Detailed mathematical representation of the integrated model can be found in [6]–[24].The flip-flop switch between the MA and VLPO groups is simulated using neuronal population modeling approach, where we average properties over the neurons in each group, rather than modeling individual neurons [27]. The evolution of the mean potential of the MA and VLPO populations are thus described by the equations
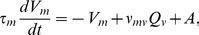
(1)

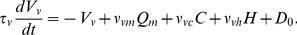
(2)


Here 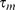 and 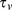 are the time delays coming from the charging of the MA and VLPO neuronal populations. The coupling strengths *ν_ij_* of the connections to the population *i* from *j* are negative for inhibitory and positive for excitatory connections (see Fig. 1A). The effects of other neuronal populations involved in modulation of the sleep-wake cycles, such as orexinergic and cholinergic nuclei, are all combined in a simplified constant input *A* to the monoaminergic neurons. The mean firing rates *Q_m_* and *Q_v_* of the neuronal populations depend nonlinearly on their mean membrane potentials:
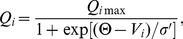
(3)where *i = m, v*, parameter *Q*_max_ is a maximum mean firing rate for the given population, 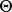 is a mean firing threshold, and 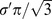 is the standard deviation of the firing threshold, which determines the slope of the sigmoid curve.

The homeostatic *H* and circadian *C* drives affect the dynamics of the VLPO as shown in Eq. (2), thereby controlling the transitions between sleep and wake (see example of dynamics in Fig. 1B). Along with the initial sleep drive level *D_0_* they contribute to the total sleep drive *D*:
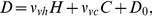
(4)with *ν_vh_>0* and *ν_vc_<0*. The total sleep drive *D* is used to estimate levels of sleepiness throughout the paper, and has units of voltage according to Eq. (2). The homeostatic drive *H* is assumed to accumulate during wakefulness and dissipate during sleep according to
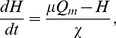
(5)where μ and χ are the time constants of the increase and decay of the homeostatic sleep pressure.

The circadian pacemaker model simulates the activity of the master circadian clock in the suprachiasmatic nucleus of the hypothalamus (SCN) under the effects of light and non-photic inputs [21]. In this model the circadian variable *x* is approximately sinusoidal and changes between −1 and 1. In our model *C* cannot be negative because it is related to the firing rate of the SCN. Therefore, we introduce the following relationship between *C* and *x,* in the same way as it was done in [6] and [24]:
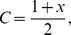
where *x* is calculated from



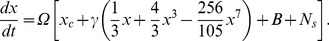
(6)The parameter *Ω* scales the period to 24 hours, and 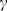 determines the stiffness of the oscillator. The complementary variable *x_c_* follows the equation
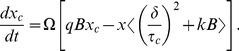
(7)


The parameters *k* and *q* are responsible for the sensitivity of the circadian pacemaker to light, and *τ_c_* is the internal circadian period. The variable *N_s_* is responsible for the non-photic influences on the phase of the circadian pacemaker, such as meals and locomotion with
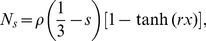
(8)where *ρ* reflects the strength of the non-photic stimulation, and *r* modulates timing of the non-photic effects in respect to the core body temperature minimum (see below). The parameter *s = 1* during wakefulness, and *s = 0* during sleep, provides state dependency of the non-photic effects.

In the St. Hilaire et al. (2007) model the photic drive *B* is assumed to be proportional to the rate *α* for the conversion of the photoreceptors in the retina from the ready state to the activated state, and to the number of receptors ready to be activated *(1-n):*

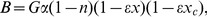
(9)where the parameters *G* and *ε* were previously adjusted to fit experimental data [21]. After being activated, the photoreceptors *n* are converted back to the ready state at a rate *β*:



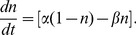
(10)The rate of conversion from the ready to activated state of photoreceptors depends on the light intensity via
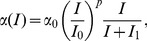
(11)where *p, I_0_*, and *I_1_* are constants, and *I* is the actual light input into the model, which is reduced during sleep due to eyelid closure:
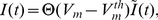
(12)where Θ is a step function and 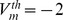 mV is a threshold mean potential above which the state is defined as wake (see [24]).

### Experimental Protocol of Czeisler et al. (1990)

In the experimental study that we simulate effects of bright versus regular light during night shifts were examined in healthy male volunteers [25]. The subjects were divided into two groups (n = 5 in a group), each undergoing either treatment protocol with bright light during the 4 nights of shift work or control protocol with regular lighting during the shifts. One of the subjects participated in both control and treatment protocols.

Each protocol consisted of one baseline week during which the men were asked to maintain regular bedtimes between 00∶00 and 08∶00, and their sleep-wake logs were recorded and verified. At the end of the baseline week the core body temperature (CBT), and several other measures were recorded during at least 27 hours of the so-called constant routine (CR) at 150 lux lighting. CR was also performed a second time after the shift work schedule was completed, and its purpose was to measure and compare endogenous circadian phase unperturbed by periodic environmental inputs, such as light/dark cycle, and influences due to periodic changes in behavior, such as sleep-wake cycle. Constant routine requires subjects to remain awake throughout, in a constant posture, restricted to very low activity levels, with nutritional intake distributed throughout day and night (for more detail on CR see [28]).

Starting the next day after these initial CR recordings the night shifts (00∶00–08∶00) were imposed for four consecutive days. In the control group lighting during the shift was set to normal indoor intensity of 150 lux. For the treatment group lighting during the shifts was bright with intensity between 7000 and 12 000 lux, and a period of constant darkness was scheduled for 8 hours starting one hour after the shift end (09∶00–17∶00). During the one hour commute home from work both groups were exposed to outdoor lighting, but the light exposure during the breaks was not controlled. CR was again performed starting on the last day of the shifts schedule following natural awakening, and the CBT was measured. The time shift of the CBT minimum (Δ*t_CBTmin_*) during the second CR relative to the first was calculated and compared between the groups.

### Implementation of the Protocol in the Model

We have reproduced the experimental protocol in the model using information provided for lighting levels during the constant routines (150 lux), shift times (150 or 12 000 lux at 00∶00–08∶00 on the shift days), and the timings of scheduled darkness (0 lux, 09∶00–17∶00 during the shift days on the treatment protocol). However, other information required for simulations, such as light exposure during baseline and during commute to and from work was not provided in the publication [25]. Thus we had to make a number of assumptions.

**Figure 2:**
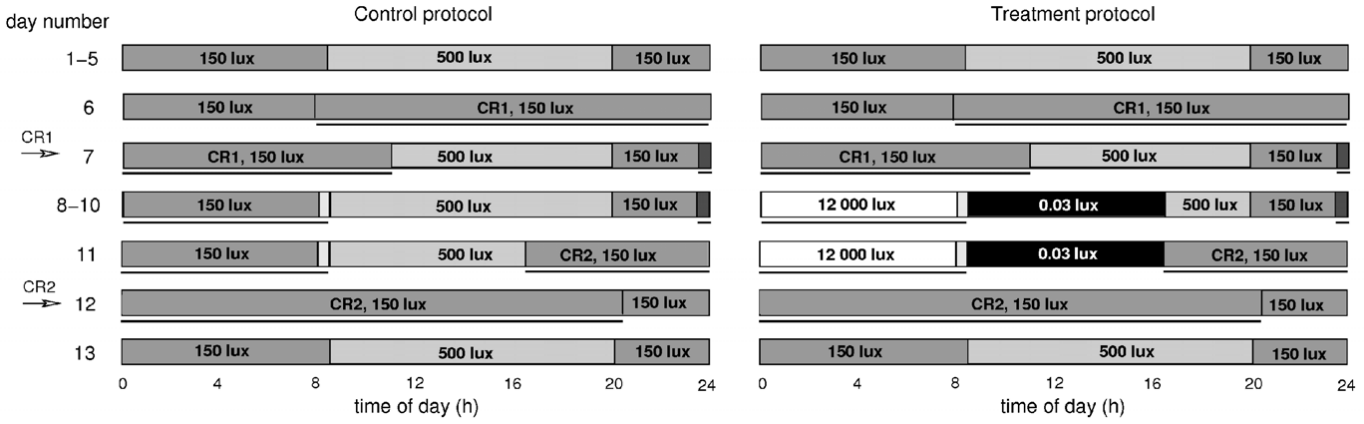
Outline of the simulated light profile and periods of forced wakefulness on the control and treatment protocols according to the experimental study [**25**]. Different shades of gray indicate different light intensity. The periods of forced wakefulness are shown with a solid line below the light intensity bars. CR1 and CR2 refer to the initial and final constant routines. Lighting during commute to and from work (days 7–11) is 25 and 5000 lux, respectively.

In particular, the experimental study did not provide information about the light profile during the baseline week. Therefore, we had to assume a reasonable light profile during the baseline and the times where lighting was not controlled in the experiment. For this purpose, we use a light profile with intensity of 500 lux between 09∶00 and 20∶00 and lower lighting of 150 lux outside these times during wakefulness, as shown for days 1–5 in Fig. 2. The intensity of 500 lux is chosen because the experimental study was done in summer, when light exposure is generally high even when subjects are at home (given that there are windows). The intensity of 150 lux is chosen between 20∶00 and 09∶00 because this is a typical value for indoor light intensity. We have also tested other realistic light profiles, including those with gradually increasing and decreasing light intensity after sunrise and before sunset respectively, and find that the major results of the paper are unaffected once the model parameters are adjusted to match experimental data (see supplementary material in Text S1, Figs S3, S4, and Table S1 for detail).

In accord with the experimental protocol constant routine on days 6–7 of the study is realised by having constant light of 150 lux and forced waking for 27 hours starting immediately after awakening on day 6 (also see red shading in Fig. 1B). After the constant routine is finished on day 7, sleep is allowed until one hour before the start of the first night shift on day 8 (see Fig. 2).

During sleep the light is gated to zero in both control and treatment groups according to Eq. (12) (see an example of the modified light profile in Fig. 1B). In some situations a low level of light input through closed eyelids can be present, but for simplicity in this study we assume that subjects sleep in dark environment. This modifies the light profile, introducing a dynamic dependence of the light on sleep-wake activity, and thereby providing a feedback on the circadian oscillator. This feedback from the behavioural state (asleep or awake) to the environment (light) and back to the circadian oscillator is an important feature of the integrated model, which is not often considered in other models.

One hour travel time is allowed for commute to and from work, and, thus, wakefulness is enforced during this time as shown in Fig. 2. Precise information about the *travel lighting* was also not provided in the experimental study; therefore, we have assumed that commute lighting corresponds to outdoor lighting profile at the time of the commute with 5000 lux in the morning (08∶00–09∶00) and 25 lux in the night (23∶00–00∶00), as illustrated with different shades of gray in Fig. 2.

Forced wakefulness during shifts, travel, and constant routines is introduced by keeping the mean membrane potentials of the MA and VLPO populations at their mean wake values: *V_m_ = 1.18 *mV and *V_v_ = *−*10 *mV (see example of *V_m_* in Fig. 1B). This allows dynamic changes of *H* and *C* according to Eqs (5)-(12) and, therefore, also tracking of the changes of the total sleep drive.

The difference between the control and treatment protocols can be seen during the days 8–11 in Fig. 2. In both cases wakefulness is enforced during the shifts, but in the control case lighting is set to 150 lux, while in the treatment case it is 12 000 lux. Additionally, darkness is enforced from 09∶00 to 17∶00 in the treatment protocol even if the subjects are not asleep. In the control protocol darkness is present only during sleep due to the abovementioned gating of light during sleep.

In both groups sleep is allowed at any time between coming home (09∶00) and going to work (23∶00) and the light during this time corresponds to the baseline light with the difference that in the treatment study darkness is enforced until 17∶00. On the last day of shift work (day 11 in Fig. 2) constant routine is again performed for 27 hours starting at 17∶00 after allowing a time to sleep. Following the CR sleep is freely allowed.

#### Timing of the core body temperature minimum

To compare the model outcomes with the experiment we calculate the timing of the core body temperature (CBT) minimum from the dynamics of the circadian variables according to the formula introduced in [21].
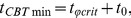
(13)


with
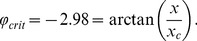
(14)


Here *t_0_* = 0.97 h is a constant, and *φ_crit_* is phase difference between the circadian variables *x* and *x_c_* of the pacemaker model, given in radians. In the experiment recordings of the *t_CBTmin_* are compared between the 7^th^ and 12^th^ days of the protocol (indicated with arrows in Fig. 2). Therefore, we use
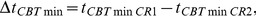
(15)where *t_CBTminCR1_* is the timing of the CBT minimum calculated according to Eq. (13) during the first constant routine, and *t_CBTminCR2_* during the second.

## Results

In the following we first demonstrate the dynamics of the model on the experimental protocol [25] with the fitted parameter values, and then use the model to investigate the dynamics in conditions that were not considered in the experiment. In particular, we examine dependence of sleepiness and adaptation to the shifts on lighting during the shifts and commute home, and on the start time of the shifts. Finally we also explore how model dynamics depend on parameter changes and show how the model can be adjusted to fit the dynamics of an individual.

### Parameters Adjustment

The parameter values for both parts of the integrated model have been calibrated previously and tested on a number of experimental studies [6]–[20]–[24]. At this default parameter values the model have demonstrated qualitative agreement with the experiment, showing much better adaptation to the treatment protocol than to the control. However, at these default values baseline sleep appeared between 23∶00 and 07∶00 instead of 00∶00–08∶00 in the experiment, and the *Δt_CBTmin_* in the control case was slightly delayed instead of being advanced. Such slight differences can be expected since the default parameter values were chosen for a case of typical sleep-wake dynamics in the population at large, whereas here the model parameters need to be adjusted to a particular set of subjects that took part in the experiment. Therefore, in order to achieve a closer fit to the experimental observations averaged across the subjects in the study we needed to find parameter sets that satisfied two conditions: (i) sleep at the baseline should appear in the range of experimentally observed mean ± SEM taking into account both control and treatment groups. This implies that sleep start time should be in the region between 23∶43 and 00∶40, as can be derived from Table 1; (ii) *Δt_CBTmin_* in response to the control protocol should likewise be in the range of the experimentally observed mean ± SEM, which, according to Table 1, gives 1.01–1.19 h. Sleep duration is set to 7.7 h and is not strongly affected by the parameters that were being adjusted (see below). We do not specifically fit the model parameters to satisfy the prediction for *Δt_CBTmin_* on the treatment protocol. Instead we use the data for treatment protocol to additionally test the validity of the adjusted parameter set by comparing model prediction with experimental observations during treatment, as shown in the next section.

**Table 1:** Comparison of the theoretical model predictions with the experimental recordings in [25] averaged across all subjects in the relevant group.

	Experiment		Model		Full-Scale Accuracy	
Measure	control	treatment	control	treatment	control	treatment
Baseline sleep onset (h:min±SEM)	00∶22±0∶18	00∶04±0∶21	00∶04		1.11%	0%
Baseline wake onset (h:min±SEM)	07∶48±0∶19	07∶33±0∶29	07∶48		0%	1.04%
*t_CBTminCR1_* (h:min±SEM)	04∶38±0∶11	05∶19±0∶23	06∶07		6.18%	3.33%
Mean sleep time during shift days (h±SEM)	5.7±0.5	7.7±0.1	4.9	7.1	3.33%	2.5%
*t_CBTminCR2_* (h:min±SEM)	03∶31±0∶56	14∶53±0∶32	05∶02	15∶05	6.32%	0.83%
*Δt_CBTmin_* (h±SEM)	1.1±0.9	−9.6±0.7	1.2	−8.9	0.42%	2.92%

SEM stands for Standard Error of the Mean. Full-scale accuracy for the match between the model and experiment is calculated as an absolute difference between the theoretically predicted value and the experimentally measured mean divided by the full period of the oscillation, which in our case is 24 hours: 100%× |*X_model_* – *X_experiment_*|/24.

**Figure 3:**
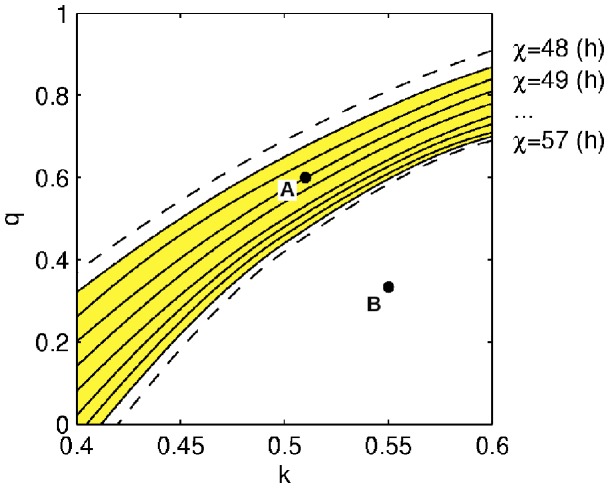
Parameter sets for fit to the experiment. Values of *k* and *q* are shown for each *χ* between 48 and 57 hours leading to a fit of the model dynamics to the experimental observations at the baseline and in response to the control protocol. Each solid line is plotted for a constant value of *χ*, with dashed lines for *χ = *48 h and *χ = *57 h representing the border cases at which baseline sleep condition is not fulfilled. Point A indicates the parameter set used in the simulation throughout the paper, and point B indicates the default values of *k* and *q* as used in [21].

We aimed to keep existing parameter values wherever possible. Thus we have chosen to adjust only three of the model’s parameters: *χ, k,* and *q*, as they constitute a minimal set required to achieve the fit. Figure 3 demonstrates the solutions for the three parameters that satisfy both fitting conditions with the light profile as shown in Fig. 2.

It is obvious from the figure that for the chosen ambient light profile there is no *χ* that would satisfy both conditions at the original values of *k = *0.55 and *q = *1/3 as used in [21] (point B in Fig. 3). Instead, there are multiple parameter sets resulting in the experimentally observed behavior for 48 h<*χ* <57 h, as indicated by the yellow area in Fig. 3. Increase of *χ* above 57 hours or its decrease below 48 hours does not allow both conditions to be satisfied with baseline sleep appearing too late for high *χ* or too early for low *χ*.

In the supplementary material (see Text S1, Figs S1 and S2) we demonstrate that different parameter sets leading to match of the model dynamics to the experimental data result in the same predictions as presented further in this manuscript. Therefore, here we choose only one representative parameter set, which is indicated with A in Fig. 3 (*χ* = 51 h, *k* = 0.51, and *q* = 0.6). All the parameter values are listed in Table 2. This set of parameters is used in all simulations except where the effects of parameter changes are examined.

**Table 2:** Parameter values of the integrated model that are used in the simulations, except where otherwise indicated.

Sleep-Wake Switch			Circadian Pacemaker		
Parameter	Value	Unit	Parameter	Value	Unit
*Q_m max_*	100	s^−1^	*Ω*	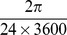	s^−1^
*Q_v max_*	100	s^−1^	*γ*	0.13	–
*Θ*	10	mV	*q*	0.6	–
*σ’*	3	mV	*k*	0.51	–
*ν_vm_*	−2.1	mV s	*δ*	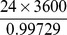	s
*ν_mv_*	−1.8	mV s	*τ_c_*	24.2×3600	s
*τ_v_*	10	s	*β*	0.007/60	s^−1^
*τ_m_*	10	s	*α_0_*	0.1/60	s^−1^
*ν_vc_*	−5.8	mV	*p*	0.5	–
*ν_vh_*	1.0	mV	*I_0_*	9500	lux
*μ*	4.4	–	*I_1_*	100	lux
*χ*	51×3600	s	*G*	37	–
*D_0_*	−11.6	mV	*ρ*	0.032	–
*A*	1.3	mV	*ε*	0.4	–
–	–	–	*r*	10×3600	s

### Simulation of the Experimental Protocol

**Figure 4:**
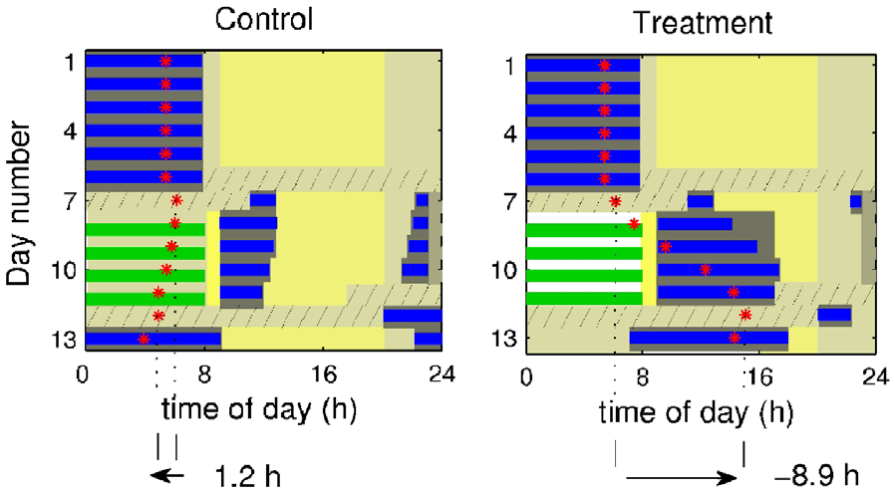
Model dynamics for the control and treatment protocols. The blue strips indicate simulated sleep and green indicate scheduled shift work. The background shades of yellow refer to light intensity at different time of the day: white – bright light of 12 000 lux, bright yellow –5000 lux during morning commute home, yellow –500 lux of the ambient light between 09∶00 and 20∶00, dark yellow –150 lux during the constant routines (hatched bars), control shifts, and ambient light when awake but outside the 500 lux zone. The light gray indicates 25 lux during commutes to work in the night, while dark gray shows darkness during sleep and scheduled darkness in the treatment protocol. Red asterisks show the timings of the core body temperature minimum (*t_CBTmin_*).

Figure 4 shows the model dynamics at the fitted parameter set on the night work protocols used in [25] with four night shifts with either 150 lux (control protocol) or 12 000 lux (treatment protocol) lighting during the shifts. During the baseline week there is no shift work, and the model demonstrates stable sleep-wake cycles, as seen in days 1–6 in Fig. 4. Red asterisks in Fig. 4 indicate the timing of the predicted core body temperature (CBT) minimum *t_CBTmin_*. During the baseline week *t_CBTmin_* = 05∶23 h, which is about 2.5 hours before awakening and is in good agreement with experimental literature showing that the CBT nadir usually appears 2–3 hours before awakening [13]. Constant routine (CR), during which wakefulness is enforced for 27 hours is applied on the 6^th^ day in both control and treatment protocols in accord with the experimental study. The predicted location of the CBT minimum during the first CR *t_CBTminCR1_* = 06∶07 h is later than *t_CBTmin_* during the baseline week, indicating the effect of the constant routine [28]. This is slightly later than the experimentally observed values, as seen in Table 1, but is in the physiologically expected range. Also note the difference between the experimental baseline *t_CBTmin_* recordings in the control and treatment groups, which are not equal because of a small number of subjects (n = 5 in each group).

After the CR is over, sleep on both protocols is freely allowed. At this point the sleep drive *D* has increased significantly due to increased homeostatic sleep pressure *H,* so sleep starts immediately after the forced wakefulness is removed. However, high circadian activity *C* during daylight hours leads to awakening after only short time of sleep, even though the homeostatic sleep pressure has not yet significantly decreased. Sleep is thus again induced briefly before waking is enforced to travel to work.

Night shift starts on the 8th day of the protocols and is repeated for 4 consecutive days, with work between 00∶00 and 08∶00. In the control case sleep is fragmented and shorter than in the treatment. This is related to the fact that the maximum of the circadian activity still appears during daytime and induces wakefulness even though the homeostatic sleep pressure has not yet fully recovered [6]. In the control case the minimum of the CBT slightly advances every day spent on the shift, while it strongly delays every day in the treatment case. Furthermore, in the treatment case sleep recovers to normal duration by the third day of the shift schedule (day 10 in Fig. 4), indicating good adaptation to night shift work, where we define good adaptation to be when sleep duration is back to normal amounts of ∼8 hours and *t_CBTmin_* appears 2–3 hours before natural awakening.

CR is again performed on the last day of shift work starting at 17∶00 on both control and treatment schedules. After the CR, sleep is freely allowed. In both control and treatment cases sleep starts immediately after the forced wakefulness is over. In the control case it continues until the next morning, indicating the lack of re-entrainment to the shift schedule. In the treatment case the sleep episode after the second CR is short, and the model is in the wake state from ∼23∶00 to about 07∶00 the next morning, indicating good entrainment to the night shift schedule.

In the experiment the measurements of the CBT minimum were compared between the first and second constant routines. Therefore, here we do the same, and calculate Δ*t_CBT_*_min_ via Eq. (15). Comparison between the model predictions and experimental outcomes is shown in Table 1. The parameters were adjusted to match the sleep and wake onset times at the baseline, and Δ*t_CBT_*_min_ on the control and treatment protocols such that the theoretical values fall in the range of the experimentally observed mean ± SEM. As the result of this adjustment also the sleep times during the shifts agree well with experiment having full-scale accuracy of 2.5% for treatment and 3.33% for control protocol as shown in Table 1. We consider an exact fit of the model to the experimental observations not necessary specifically because the number of subjects in the experiments was low, and different subjects (except one) participated in the control and treatment protocols. Therefore, it cannot be expected that their dynamics can be precisely described with the same parameter set.

We have also compared mean sleep drives during the shift times 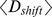 on the 1^st^ and 2^nd^ constant routines, i.e., average sleep drive *D* between 00∶00 and 08∶00 on days 7 and 12, in order to estimate the effects of control and treatment protocols on sleepiness. This measure was not assessed in the experiment, so it cannot be directly compared with the data provided in [25]. In the control case 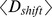 has increased from 4.3 mV to 6.1 mV, indicating increasing risk of accidents on such a shift protocol. In contrast, on the treatment protocol mean sleep drive has decreased from 4.3 mV to −0.12 mV, indicating an improved alertness compared to the beginning of the shift schedule. Note that the values of 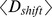 do not yet have an exact experimentally measurable correlate, but in some cases can be compared to measures of subjective alertness [23] or performance and vigilance tests. In this study 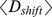 is used as a qualitative indication of sleepiness levels, with negative values indicating lower sleepiness.

### Model Predictions

Since the model dynamics agree well with the experimental observations we can use it to explore and predict adaptation to night shifts in other conditions and to design shift schedules with lower sleep drive.

#### Effects of treatment light intensity

**Figure 5:**
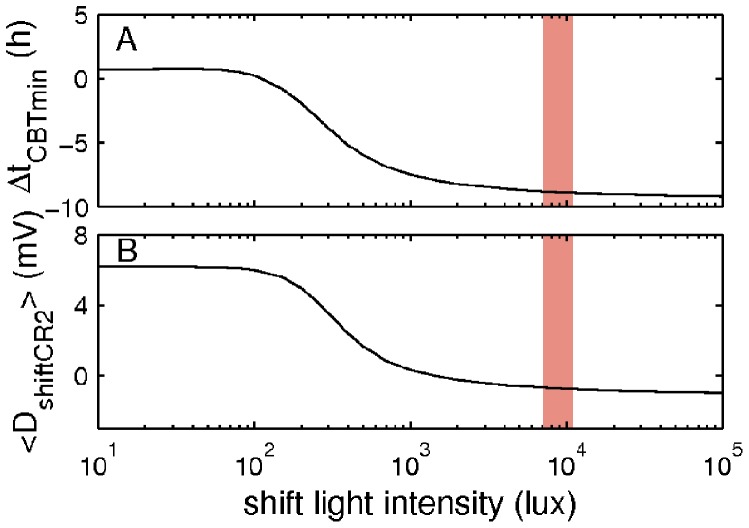
Effects of the shift light intensity on adaptation to night work. Panel A demonstrates dependence of the simulated Δ*t_CBT_*_min_ on shift light intensity in the treatment case, and panel B shows the dependence of the mean shift sleepiness during the second constant routine 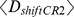. Red shading indicates the range of light intensities used in the experiment.

Lighting intensities used at workplaces generally vary between about 10–50 lux for patrolling police officers or truck drivers on night shifts and up to 350–500 lux in offices, clinics, and factories. The treatment light intensity used in the experiment (7000–12 000 lux) is high, and would be expensive, and sometimes impossible, to use at workplaces. Therefore, in order to see whether light intensity can be reduced we examine how different shift light intensities affect entrainment to the shifts in the treatment conditions. Figure 5 demonstrates how Δ*t_CBT_*_min_ and mean shift sleep drive on the treatment protocol change depending on the lighting intensity during the shifts, while all other conditions are kept the same as in Fig. 4.

According to Fig. 5 higher light intensities lead to a longer delay of the CBT minimum and lower values of the mean sleep drive during the shift time. However, the dependence of Δ*t_CBT_*_min_ on the lighting is nonlinear and the slope of the Δ*t_CBT_*_min_ change is significantly reduced for *I* ≥1000 lux. The difference in Δ*t_CBT_*_min_ between 1000 lux and the 12 000 lux used in the experiment, is 1.5 h, and still leads to quite good adaptation and reduced sleep drive. The light intensity of 3000 lux, which is still fourfold lower than that used in the experiment, leads to an only half an hour smaller shift of the CBT minimum compared to 12 000 lux (−8.4 h vs. −8.9 h). Given that in reality shift workers are also affected by various random inputs, such a difference can be considered as minor. On the other hand, light intensities below 100 lux lead to high sleep drive and phase advance of the temperature rhythms and, thus, advance of the circadian pacemaker *(*Δ*t_CBT_*_min_ >0). According to Fig. 5, shift light intensity of 150 lux leads to Δ*t_CBT_*_min_ = −0.79 h. However, in the control case, where the same shift light intensity is used, Δ*t_CBT_*_min_ = 1.2 h (see Fig. 4 and Table 1). This demonstrates the effect of the scheduled darkness introduced in the treatment protocol during the rest time at home, which is absent in the control case (see Fig. 4 and Methods).

#### Effects of commute light intensity

It has long been demonstrated that, in order to improve entrainment to night shift work, one has to reduce the amount of light exposure during commute home in the morning hours, when outdoors light intensity is generally high (for a review see [5]). This can be done, for example, by wearing sunglasses with low transmission coefficients on the way home or choosing subway commute instead of a walk outdoors. To explore this in the model we examine the dependence of Δ*t_CBT_*_min_ and 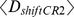 on morning commute light intensity in control and treatment cases.

**Figure 6:**
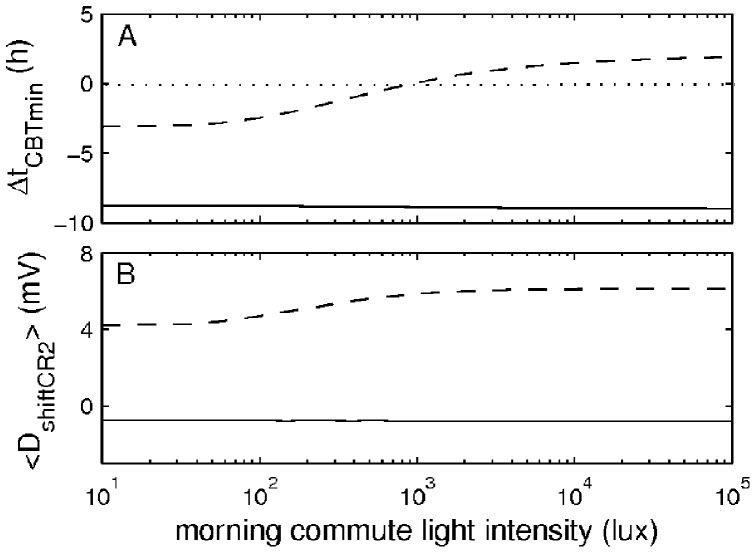
Effects of the morning commute light on adaptation in control (dashed line) and treatment (solid line) protocols. Panel A demonstrates dependence of the simulated Δ*t_CBT_*_min_ and B of the mean shift sleepiness during CR2 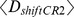 on the intensity of the morning commute lighting. The dotted line indicates zero level for the shift of CBT minimum.

The results in Fig. 6 show that the intensity of commute light does not have much effect on the response in the treatment protocol (solid lines in Fig. 6A and B). However, in the control case decrease of the commute lighting below 1000 lux leads to the transition from advance of *t_CBTmin_* to delay. Consequently, also the mean shift sleep drive is decreased. At a commute lighting of 10 lux the mean shift sleep drive decreases almost to its initial level 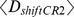 = 4.2 mV, with 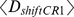 = 4.3 mV.

#### Effects of shift onset time

It is obvious that different start times of the shift will result in different adaptation to the schedule due to phase dependency of the circadian response to light (see [6]–[29]). This means that best and worst start times of the night shifts can be found and used to provide additional information for better scheduling of work. Therefore, here we study the effects of shift start time on adaptation to shifts on the given protocol. Note, that the commute lighting is also changed depending on the shift start time according to natural summer light profile with sunrise at 05∶30 and sunset at 20∶30. A minimum light intensity of 25 lux is set for night commutes and a maximum intensity of 5000 lux for morning/day commutes.

**Figure 7:**
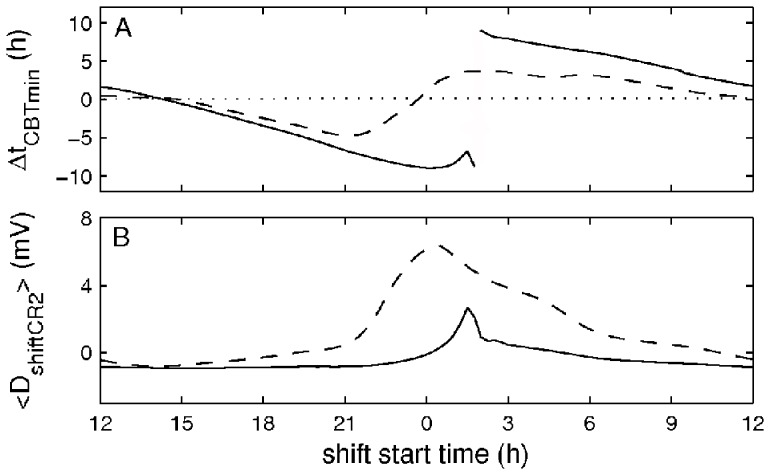
Effects of the shift start time on adaptation to the schedule. Panel A demonstrates dependence of Δ*t_CBT_*_min_ on the start time of the shifts on the schedule, and panel B shows the dependence of the mean shift sleepiness during the second CR. Results for the treatment study are shown with solid lines, and for the control with dashed lines. The dotted line indicates zero level of the time shift of CBT minimum.

Figure 7 illustrates the dependence of Δ*t_CBT_*_min_ and 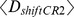 on the shift start time. The first important observation is that in both control (dashed line) and treatment (solid line) cases, there is a transition between delay and advance of *t_CBTminCR2_* for night shift schedules. For the control case it is observed for shifts starting near 23∶45, and the transition is smooth – a so-called type 1 transition [30]. In the treatment case the transition happens for shifts starting near 01∶45 and is abrupt (type 0), switching from Δ*t_CBT_*_min_ = −9.88 h to Δ*t_CBT_*_min_ = +9.2 h. According to Fig. 7B these are also the shift schedules with highest mean sleep drive.

The type 0 transition of the circadian phase as seen on the treatment protocol has also been observed on longer shift schedules with lower light intensity [6]. There the highest sleep drive was likewise observed on the shifts starting close to the transition point. This correlation is explained by the fact that at the transition point the circadian maximum is located in the middle of the allowed rest time, which leads to minimal sleep times, increased homeostatic pressure *H,* and thereby increased sleep drive *D*.

The phase transition point appears when the middle of the light exposure is close to the maximum of the circadian oscillation. For a symmetrical oscillation this would be an unstable point where the system can either advance or delay, depending on an input, or stay unaffected. In our case the circadian oscillator is forced to delay due to an asymmetric shape of the circadian oscillation. This results in the system delaying by 16 hours, instead of advancing by 9 hours on the shifts starting immediately after the transition point in Fig. 6. As shown before, this also leads to the longest adaptation time [6].

According to Fig. 7B, an earlier start of the night shifts significantly decreases sleepiness, especially in the control case. For example, the shift schedule with work starting at 21∶00 leads to 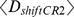 = 0.46 mV, while for the midnight schedule used in the experiment 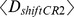 = 6.1 mV. Notably, shift schedules starting in the afternoon result in similar sleep drive for control and treatment cases. This happens because these schedules do not require strong circadian re-entrainment.

**Figure 8:**
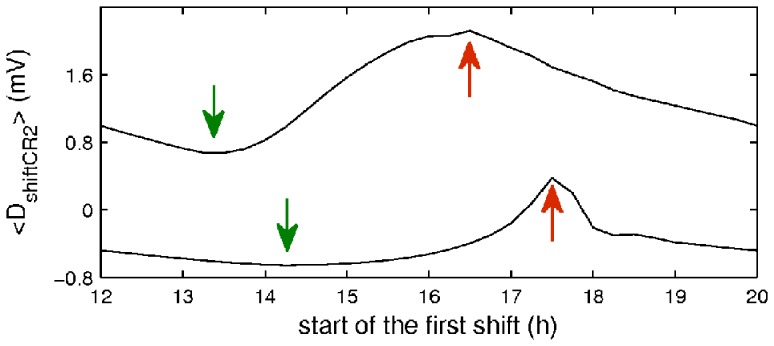
Effects of the shift onset for a schedule of 3 shifts covering an entire day on the mean sleepiness. The dashed and solid lines indicate the mean shift schedule sleepiness in the control and treatment cases. Green arrows refer to the shifts’ start time with the lowest average shift sleep drive (optimal), and red arrows to that with the highest average shift sleep drive (worst).

In industry, work often has to be performed around the clock. The results in Fig. 7 allow us to estimate which around the clock schedule of 8 h shifts in the same light conditions as used in our protocols would have the highest and the lowest sleepiness across all shifts, and thus would be the best or the worst for this particular conditions. In order to cover the entire 24 hours of a day there should be three 8 h shifts, each starting when the previous one is over. Figure 8 demonstrates an average shift sleep drive across such a schedule of three shifts depending on the start time of the first shift, which we have chosen between 12∶00 and 20∶00. Such shift schedule corresponds to 3 independent groups of workers each performing 4 shifts after a week of stable baseline sleep-wake cycles, as in our protocols. Note, that this is not a rotating shift schedule where each worker undergoes rotation, but a schedule where different workers do different shifts.

As Fig. 8 suggests for the control case the schedule with the lowest mean sleep drive is the one with the shifts starting at 13∶30, 21∶30, and 05∶30 (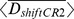 = 0.68 mV), while highest mean sleep drive is observed on the schedule with shifts starting at 16∶30, 00∶30, and 08∶30 (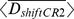 = 2.1 mV). Likewise, for the treatment case the best schedule is 14∶15, 22∶15, and 06∶15 (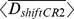 = −0.65 mV), whereas the worst one is 17∶30, 01∶30, and 09∶30 (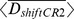 = 0.38 mV). However, the mean sleep drive on the worst treatment schedule is still lower than that on the best control schedule (0.38 vs 0.68), demonstrating the beneficial effects of the higher light intensity during work.

In both cases the schedules containing a shift close to the transition point have highest mean sleep drive, whereas the lowest mean sleep drive is observed on the schedules starting nearly 3 hours before the worst ones. Furthermore, the 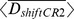 curve in the interval ±2 h around the minimum point is fairly flat in the treatment case (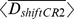 increases by 0.15 mV at −2h, and 0.18 mV at +2 h compared to the minimum), whereas the minimum is narrower in the control case (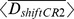 increases by 0.45 mV at −2h, and 1.2 mV at +2h compared to the minimum). This suggests that in the treatment conditions the shifts do not have to be scheduled precisely near the minimum point to achieve low mean sleep drive, while in the control case scheduling needs to be more precise. Furthermore, in both cases it is beneficial to schedule the shifts earlier since the increase of mean sleep drive is slower in this direction.

### Model Dynamics at Different Parameter Values

In the simulations in previous sections we have used the parameter set fixed to match the experimental results from [25] that were averaged across all the subjects. However, individuals normally differ in their circadian and homeostatic parameters, and are thus expected to have different responses to the same shift schedule. For example, Δ*t_CBT_*_min_ for the subjects in the control protocol in [25] varied between approximately −1.2 and 3 h, with 2 out of 5 subjects demonstrating circadian delay, and 3 demonstrating advance. Likewise in the treatment group the variation was approximately between −8.2 h and −12.5 h, with 3 of the subjects delaying by about −9.2 h, one showing the shorter delay of approximately −8.2 h and one showing a very long delay of −12.5 h (the data are derived from Fig. 2 in [25]). Therefore, in order to adjust the model to individuals’ dynamics different parameter sets should be used.

To study how the different response to the experimental protocol can be achieved we examine how change of the model’s parameters affects the dynamics. In particular we estimate the effects of (i) the parameter *χ,* which is a time constant for accumulation of homeostatic sleep pressure during wake; (ii) the parameter *k* that regulates circadian sensitivity to light and is responsible for the fulfillment of the Aschoff rule postulating that diurnal animals (*k*>0) delay in constant light conditions [31]; (iii) the parameter *q*, which is also responsible for the sensitivity of the circadian pacemaker to light; and (iv) the internal circadian period *τ_c_*. The first three parameters have been chosen because they were initially tuned to achieve a good fit to the averaged experimental data, and the internal circadian period was chosen because this characteristic often differs in people and can significantly affect response to night shifts.

Since we do not have detailed information about sleep times of different subjects at the baseline, but know that they were asked to sleep between 00∶00 and 08∶00, we have to assume that this is their baseline sleep time. However, we found that change of any single control parameter leads to change of the baseline sleep time. Therefore, in order to keep baseline sleep times constant, and in line with the previous simulations, we have to balance such a change by tuning another parameter. In this way we always change two parameters and have the same baseline conditions, but different responses to shift protocols.

**Figure 9:**
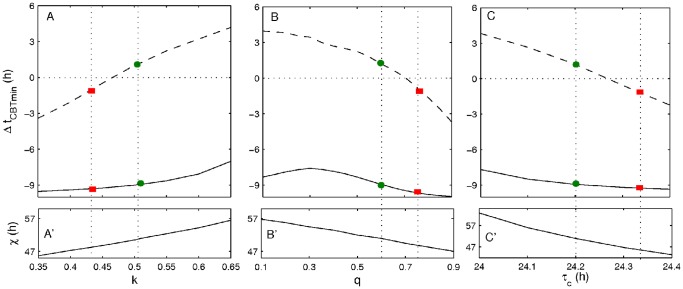
Dependence of the shift of the core body temperature minimum (Δ*t_CBT_*_min_) in the control and treatment cases on the parameter changes. Panel (A) demonstrates how Δ*t_CBT_*_min_ changes with tuning of the parameters *χ* and *k*, with panel (A′) illustrating the values of the parameters at which the results in (A) are obtained. Panels (B) and (B′) demonstrate the same dependencies obtained for the pair of parameters *χ* and *q*, and panels (C) and (C′) for the parameters *χ* and *τ_c_*. Dashed lines indicate the results obtained in the control case, and solid line shows Δ*t_CBT_*_min_ obtained in the treatment case. The plots for each of the parameter pairs are made at fixed values of other parameters according to the values in Table 2. The vertical dotted lines indicate the values of parameters that are used to simulate the experimental results. The green circles correspond to the parameter values and Δ*t_CBT_*_min_ as used in Figs. 1,4–8. The red squares indicate the Δ*t_CBT_*_min_ observed for one subject in [25] who participated in both control and treatment protocols, and indicate the parameter values that can be used to achieve these dynamics.

In this study we use the homeostatic time constant *χ* to adjust the baseline sleep time when one of the circadian parameters (*k, q*, or *τ_c_*) is tuned. Increase of *χ* normally leads to a later sleep onset, while its reduction yields an earlier one. Figures 9A’, B’, and C’ demonstrate the values of *χ* at which sleep at the baseline is achieved between 00∶00 and 08∶00 when varying one of the parameters *k*, *q,* and *τ_c_*. For each of the figures the rest of the parameters are kept constant at the values corresponding to the fit to the average experimental case as shown in Table 2.

Increase of the parameter *k* alone leads to earlier sleep onset; therefore, in order to keep sleep during baseline week the same in all simulations the parameter *χ* must be increased to delay the sleep onset accordingly. Figure 9A’ demonstrates which pairs of *χ* and *k* lead to the same baseline conditions (sleep between 00∶04±2 min and 07∶48±2 min to have better agreement with the fit in Table 1), while the rest of the parameters are kept constant. The increase of both *k* and *χ,* as shown in Fig. 9A, leads to increase of the difference between the Δ*t_CBT_*_min_ for the treatment and control studies. At *k = *0.466 *(χ = *49.3 h) the control protocol does not shift the CBT minimum. Below this value the system shows phase delay of the circadian pacemaker, and phase advance above this point. The response to the treatment study is more stable, demonstrating strong phase delay of the circadian pacemaker across all the *k* values, with slight decrease of Δ*t_CBT_*_min_ at higher values of *k* and *χ.*

Increase of the parameter *q* from the original value of 0.3, used in [21], leads to the opposite trends. First an increase of *q* has to be compensated by decrease of *χ* in order to achieve the same baseline sleep conditions. Second, increase of *q* leads to stronger delay of the *t_CBTmin_* in both control and treatment cases. In the control case, increase of *q* from 0.1 to 0.9 leads to a monotonous decrease of Δ*t_CBT_*_min_ from advancing to delaying values, while in the treatment case there is a maximum (smallest delay) of Δ*t_CBT_*_min_ at *q = 0.3.*

Finally, change of the internal circadian period also leads to the dynamics opposite to that with the change of *k*. In order to compensate for the delay of the sleep onset at higher values of *τ_c_*, the homeostatic time constant *χ* has to be reduced, as seen in Fig. 9C’. With increase of *τ_c_*, Δ*t_CBT_*_min_ is decreasing for both control and treatment protocols. However, the response in the treatment protocol is much less pronounced than in the control. By choosing appropriate parameter values based on Fig. 8 one can adjust the model to demonstrate dynamics similar to that of individual subjects. For example, the Czeisler et al. [25] study included data for one subject who participated in both control and treatment protocols. The results of the CBT minimum shift for this subject were: Δ*t_CBT_*_min *control*_ = −1.2 h, and Δ*t_CBT_*_min *treatment*_ = −9.2**h (see Fig. 1 in [25]). In the model these results can be reproduced by tuning different parameters at the same baseline conditions, as demonstrated with red squares in Figs. 9A, B, and C. For example, the result achieved by tuning the parameters to *k = *0.43 and *χ* = 48.2 h is Δ*t_CBT_*_min *control*_ = −1.2 h, and Δ*t_CBT_*_min *treatment*_ = −9.3**h. Similar outcomes are achieved by keeping *k* constant but changing either *q* and *χ* accordingly to *q* = 0.78, *χ* = 48.5 h or *τ_c_* and *χ* to *τ_c_* = 24.34 h, *χ* = 45.3 h. Note that the parameter values are always kept within a physiologically justified range, e.g., values of *χ* and *τ_c_* can be measured experimentally. The values of *k* and *q* cannot be so easily measured, but are estimated from the fit to experimental data [21].

## Discussion

We have demonstrated how our integrated model can be applied to examine adaptation to night work and to optimize shift schedules and conditions. In particular, the model predicts weak adaptation and high sleepiness on the night shifts schedule in low light conditions (150 lux) and good adaptation with decreased sleepiness for the case of high lighting during the shifts (12 000 lux). This result is observed even without specific parameter adjustment, confirming that qualitative model predictions can be trusted even with the default parameter set. We find that the parameters can be adjusted to fit the particular set of subjects in the experimental study, thus allowing quantitative predictions for this group. This can be done with a number of different parameter sets, but these parameter combinations lead to similar predictions. Further, using the adjusted model, we have demonstrated its utility for prediction of shift times resulting in lower sleep drive and faster adaptation in lower light conditions. For example, we find that treatment light intensity can be reduced to 3000 lux, while still leading to similar adaptation as observed at 12 000 lux, and that starting shifts on this schedule at 21∶00 instead of 00∶00 significantly decreases sleep drive in both control and treatment cases. Finally, we have demonstrated that the model parameters can be adjusted to fit the dynamics of specific individuals on this protocol, and that individuals with the same baseline sleep characteristics can have drastically different response to shift work.

Due to the low number of subjects in the experimental study [25] to which the model was fitted the predictions cannot be generalized to the population at large. However, it can be expected that the predictions will hold qualitatively, especially since the findings reported in [25] were further confirmed by other groups, e.g. [5]–[32]. The predictions made in the present paper can and should be examined experimentally. In particular the estimation of optimal and worst shift start times can be examined in a study where subjects first undergo a protocol similar to that of Czeilser et al. in order to fit the model to the individuals knowing their baseline activity and response to perturbation. Second, predictions for response to other (optimal or worst) shift times should be made using the adjusted model and tested in experiment. Such a study would be particularly useful for further validation of the model.

It is noteworthy that the model parameters cannot be further calibrated based on the existing published experimental shift work studies, because, as far as we are aware, they do not provide complete information required for simulations. In the present study, for example, we had to make assumptions for ambient light profile, which affects the choice of parameter sets that match experimental observations. However, our study also indicates that such precise information may not even be needed, since the predictions for optimal and worst shift conditions remain similar for different examined light profiles once the parameters are adjusted (as shown in supplementary material). This may not be the case for other predictions and light conditions and has to be further investigated. Therefore, in the future it will be of advantage to further narrow down the parameter ranges provided a more complete experimental data for large number of subjects. We elaborate on the key findings of the present study below.

### Treatment Light Intensity

The model predicts that the light intensity during the shifts in the treatment protocol can be reduced to ≈3000 lux, while giving practically the same adaptation as that in the case of 12 000 lux. Lower lighting level at the workplace that still allows good adaptation to shifts is highly preferable because it makes less damage to the retina, and is cheaper in terms of electric power. The above results support experimental findings of Boivin et al. [32], who showed that exposure to light of intensity about 3500 lux during night shifts is sufficient for re-entrainment of workers. This confirms the utility of the model for prediction of optimal light conditions to allow good adaptation to shift schedules.

### Morning Commute Light Intensity

Our simulations have demonstrated that adaptation to the night shift schedule in the control case strongly depends on the light intensity during the morning commute home from work, with lower light intensity leading to better adaptation and lower mean sleep drive. This is in good agreement with experimental studies showing that use of sunglasses or other ways of reducing light exposure during morning commute improves shift workers’ adaptation (for reviews see [5]–[14]). This, again, supports the validity of the model for predictions of adaptation to shift work.

Furthermore, given that in the experiment the lighting during commute home was not controlled, different subjects could have been exposed to very different light; e.g., some may have walked home, while others took the subway. This factor could have contributed to the broad distribution of responses to the control protocol in the experimental study. The fact that response to the treatment protocol does not significantly change with different commute lighting, also supports this idea, since in the experiment the distribution of responses to the treatment protocol was narrower, although we stress that the number of subjects was small.

Another commute factor affecting adaptation to shift work is the duration of travel. In this study we have considered a generalized case where people are allowed 1 hour for their commute, and are not allowed to sleep while traveling. However, it is clear that those who live closer or farther from work would need different commute time, and those using public transport may doze off during travel, as opposed to those driving a car. Based on our study we can predict that lower commute times would have two effects: (i) reduce outdoors light exposure, which we have shown to be beneficial for adaptation to shift work (Fig. 6), and (ii) allow longer sleep time, especially during the first days on night shifts before the circadian process is sufficiently strong to induce awakening. This should allow faster recovery of sleep debt, and thereby decrease sleepiness in the short term. Longer commute times may lead to the opposite effects, but need to be studied in more detail, because the nonlinear nature of the human phase response curve to light can lead to complex interdependencies.

### Shift Start Time

In our study night shifts have been demonstrated to be the worst in terms of mean sleep drive, which agrees well with experimental data [3]–[33]. However, we find that scheduling of such shifts to start at 21∶00 instead of 00∶00 in both control and treatment cases significantly reduces sleep drive. In the treatment case, shifts starting between 01∶00 and 03∶00 lead to the highest sleep drive, whereas for the schedule with shifts starting at 01∶45, sleep drive on the treatment schedules is close to that on the control protocol.

Given natural individual variability, it can be expected that the shift starting times leading to phase transitions in control and treatment cases will be slightly different for different subjects. Importantly, in the control case the critical shift start time is 23∶45, which is very close to the shift start time used in the experiment (00∶00). This can contribute to the variety of individual results observed in the experiment, where two out of five subjects demonstrated delay of the CBT minimum, while the other three advanced. Such variety was not observed in the treatment case because the shift start time used in the experiment was quite far from the critical point of the treatment case (01∶45). However, one of the subjects had demonstrated an unusually large shift in the *t_CBTmin_* of −12.5 hours (see Fig. 2 of [25]). It is possible that internal characteristics of this subject were such that his phase transition happened closer to 00∶00, and thus the circadian pacemaker was forced to delay by a longer time in order for the *t_CBTmin_* to appear earlier in the day [6]. It would be interesting to test experimentally whether scheduling of the shifts to start around 01∶45, but otherwise staying on the same experimental treatment protocol, would lead to the wider variety of individual responses (with either delay or advance of the CBT minimum) predicted by our simulations.

Such a critical point for shifts starting time exists for every light protocol, according to the human phase response curve. Although the transition can be of either type 1 (as in the control case) or 0 (as in the treatment case), it nevertheless leads to an increased sleep drive, and therefore increases risk of work-related injuries and reduces the overall quality of life. Thus, it is important to know where the critical point is located for specific work conditions in order to avoid scheduling of the shift onsets near this time. This is specifically the case for the control protocol, where variations of sleep drive depending on the shift start time are much larger than in the treatment case where the mean sleep drive curve is flatter (see Figs 7,8). This also means that more freedom can be allowed when choosing shift start time on the treatment protocol than on the control in order to minimize sleep drive levels.

Using the information obtained from Fig. 7 we have also predicted the best and worst start times for schedules covering the entire 24 hours of the day with 3 independent groups of workers performing 4 shifts after being on normal day schedules prior, as demonstrated in Fig. 8. For businesses using such schedules these predictions are of help for more efficient organization of the shifts with improved overall performance of the workers. However, it needs to be remembered that in this study the effects of weekends, which can affect the results in longer term, were not considered. Also, rotating shifts, where a person works a certain number of days on each morning, day, and night shifts, are more widely used.

### Model Dynamics at Different Parameter Values

Finally, because of natural variability among subjects, we have examined how the model dynamics depend on model parameters. Since we have aimed to keep the original parameter values wherever possible, here again we have chosen to change parameters that were needed for initial fit of the model to experimental data: *χ*, *k,* and *q*. Additionally we have chosen to study how the model dynamics depend on the internal circadian period *τ_c_* because this parameter is often different in individuals.

We have demonstrated that the model can be adjusted to required individual dynamics by means of different slight parameter changes. However, the ranges of the parameter values from which we can choose are not free, but determined by physiological characteristics, many of which can be directly measured experimentally, such as *χ* and *τ_c_*, while other are constrained based on the model dynamics, such as *k* and *q* [20]–[21]. It is important to note that the changes in the model parameters required to mimic individual dynamics do not necessarily reflect the real physiological differences among individuals. For example, as we have shown in Fig. 9 a difference in the value of the light sensitivity parameter *k* can lead to the same dynamics as difference in the internal circadian period *τ_c_*. This demonstrates flexibility of the integrated model, but it also means that the data provided by the protocol in [25] are insufficient to determine the parameters for individual subjects more precisely. Different experiments must be done in order to further narrow down the possible parameter ranges; For example, the homeostatic time constant *χ* can be estimated from polysomnographic recordings [7], while *τ_c_* can be measured in forced desynchrony protocols [34].

The model also suggests that the individuals showing the same dynamics at the baseline can have significantly different response to the shift schedules. In other words this means that people with seemingly the same chronotype in normal conditions can have very different responses when sleep-wake cycles are perturbed by shift work. At the same time, we have demonstrated that whenever the sleep timing at baseline and response to the midnight shift are both preserved, even different parameter sets lead to the same overall predictions (see supplementary material). This indicates that the knowledge of chronotype alone may be insufficient to estimate how well people adapt to shifts, but the knowledge of chronotype together with the data on response to perturbation, such as night shift, can be enough to make predictions for individuals. In this study baseline sleep was adjusted to appear between 00∶00 and 08∶00 which corresponds to midsleep time around 04∶00, which is one of the most common chronotypes [35]. However, other chronotypes, as well as other intrinsic properties such as habitual sleep duration, also need to be examined, since they may have strong effects on adaptation to shift work.

### Summary

In the present study we have demonstrated that the integrated model can be adjusted to quantitatively match experimental shift work data, and have shown how adaptation to shift work can be improved by changing such external factors as lighting and timing of the shifts. However, it has to be noted that there is an infinite number of possible conditions affecting adaptation, including different shift work schedules, light profiles, and intrinsic workers’ sleep properties. Obviously, they cannot all be examined in advance even using a mathematical model. However, a physiologically based model can be calibrated on a number of specific core studies, and then applied to other conditions as desired by users.

## Supporting Information

Figure S1 **Parameter sets satisfying the conditions for fit to the experiment.** Values of *k* and *q* are shown for each *χ* between 48 and 57 hours leading to a fit of the model dynamics to the experimental data at the baseline and in response to the control protocol. Solid lines are plotted for constant values of *χ*, with dashed lines for *χ* = 48 h and *χ* = 57 h representing the border cases at which baseline sleep condition is no longer fulfilled. Point A indicates the parameter set used throughout the paper, B - default values of *k* and *q* as used in [21], C (*χ* = 49 h, *k* = 0.5, *q* = 0.65), D (*χ* = 56 h, *k* = 0.52, *q* = 0.51), E (*χ* = 53 h, *k* = 0.48, *q* = 0.44), F (*χ* = 50, *k* = 0.54, *q* = 0.71) – indicate the parameter sets used in simulations in Fig. S2.
(TIF)

Figure S2 **Dependence of adaptation on shift start times for different parameter sets allowing fit to the experimental data.** The colors of the lines correspond to the parameter sets from Fig. S1: A-black, B-green, C-red, D-orange, E-blue. Note, that dependencies for set A are the same as in Fig. 7 of the paper. Dashed lines refer to the results for control protocol and solid lines to the treatment protocol.
(TIF)

Figure S3 **Examples of the examined ambient light profiles.** Note, that these light profiles are additionally modified by the dynamics of the model, as the light is gated during sleep, and are further modified during the shift protocols
(TIF)

Figure S4 **Dependence of adaptation on shift start times for different ambient light profiles and parameter sets according to Table S1.** The colors correspond to the different light profiles: red – profile 1, black – profile 2, green – profile 3, blue – profile 4. The black line for light 2 is the same as in Fig. 7 of the paper. Dashed lines refer to the results for control protocol and solid lines to the treatment protocol.
(TIF)

Table S1 **Parameter values corresponding to the ambient light profiles shown in Fig. S3.** The parameter sets are chosen in accord with conditions outlined in “Parameter adjustment” section. Profile 2 and the corresponding parameter set (set A in Fig. S1) are the ones that are used throughout the paper.
(DOC)

Text S1 **Supporting information on adjustment of the model parameters and model dynamics at different ambient light profiles.**
(DOC)
