# Supplementary figures and images for: Adaptation to Shift Work: Physiologically Based Modeling of the Effects of Lighting and Shifts’ Start Time
Source: PLoS One. 2013 Jan 4;8(1):e53379. doi: 10.1371/journal.pone.0053379 (PMC3537665; doi:10.1371/journal.pone.0053379)

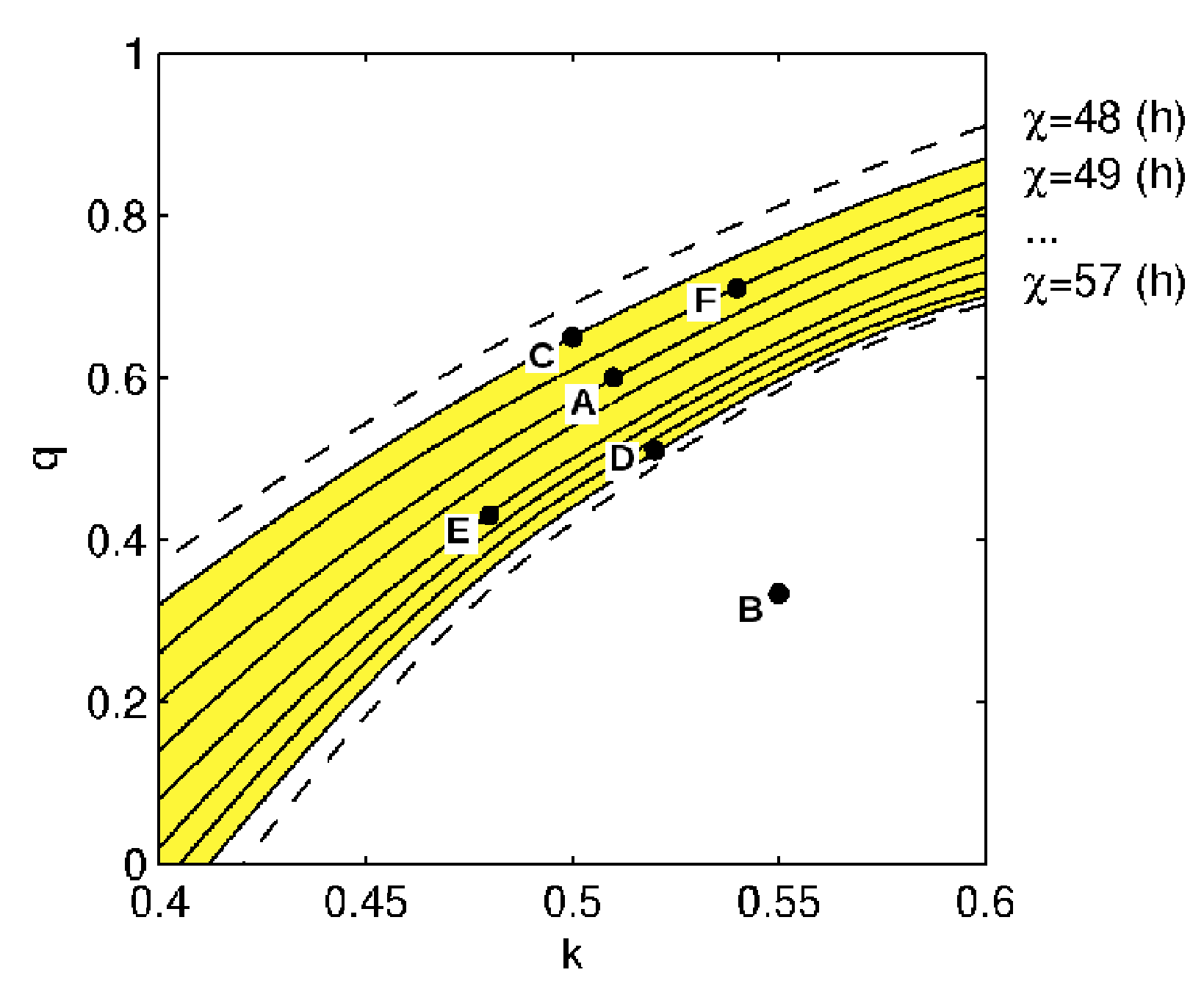

Supplement: Figure S1 — Parameter sets satisfying the conditions for fit to the experiment. Values of k and q are shown for each χ between 48 and 57 hours leading to a fit of the model dynamics to the experimental data at the baseline and in response to the control protocol. Solid lines are plotted for constant values of χ, with dashed lines for χ = 48 h and χ = 57 h representing the border cases at which baseline sleep condition is no longer fulfilled. Point A indicates the parameter set used throughout the paper, B - default values of k and q as used in [21], C (χ = 49 h, k = 0.5, q = 0.65), D (χ = 56 h, k = 0.52, q = 0.51), E (χ = 53 h, k = 0.48, q = 0.44), F (χ = 50, k = 0.54, q = 0.71) – indicate the parameter sets used in simulations in Fig. S2. (TIF) [file pone.0053379.s001.tif]

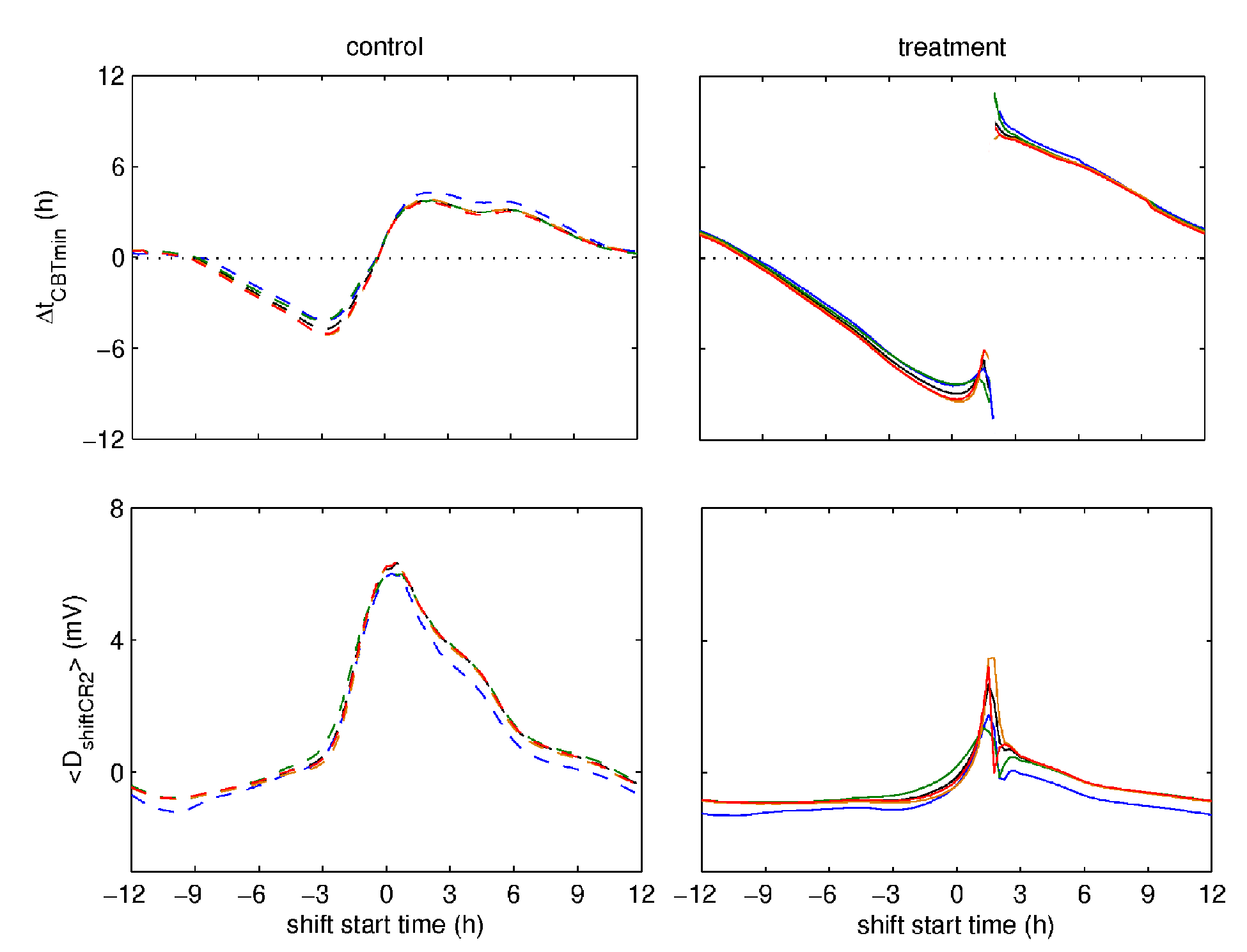

Supplement: Figure S2 — Dependence of adaptation on shift start times for different parameter sets allowing fit to the experimental data. The colors of the lines correspond to the parameter sets from Fig. S1: A-black, B-green, C-red, D-orange, E-blue. Note, that dependencies for set A are the same as in Fig. 7 of the paper. Dashed lines refer to the results for control protocol and solid lines to the treatment protocol. (TIF) [file pone.0053379.s002.tif]

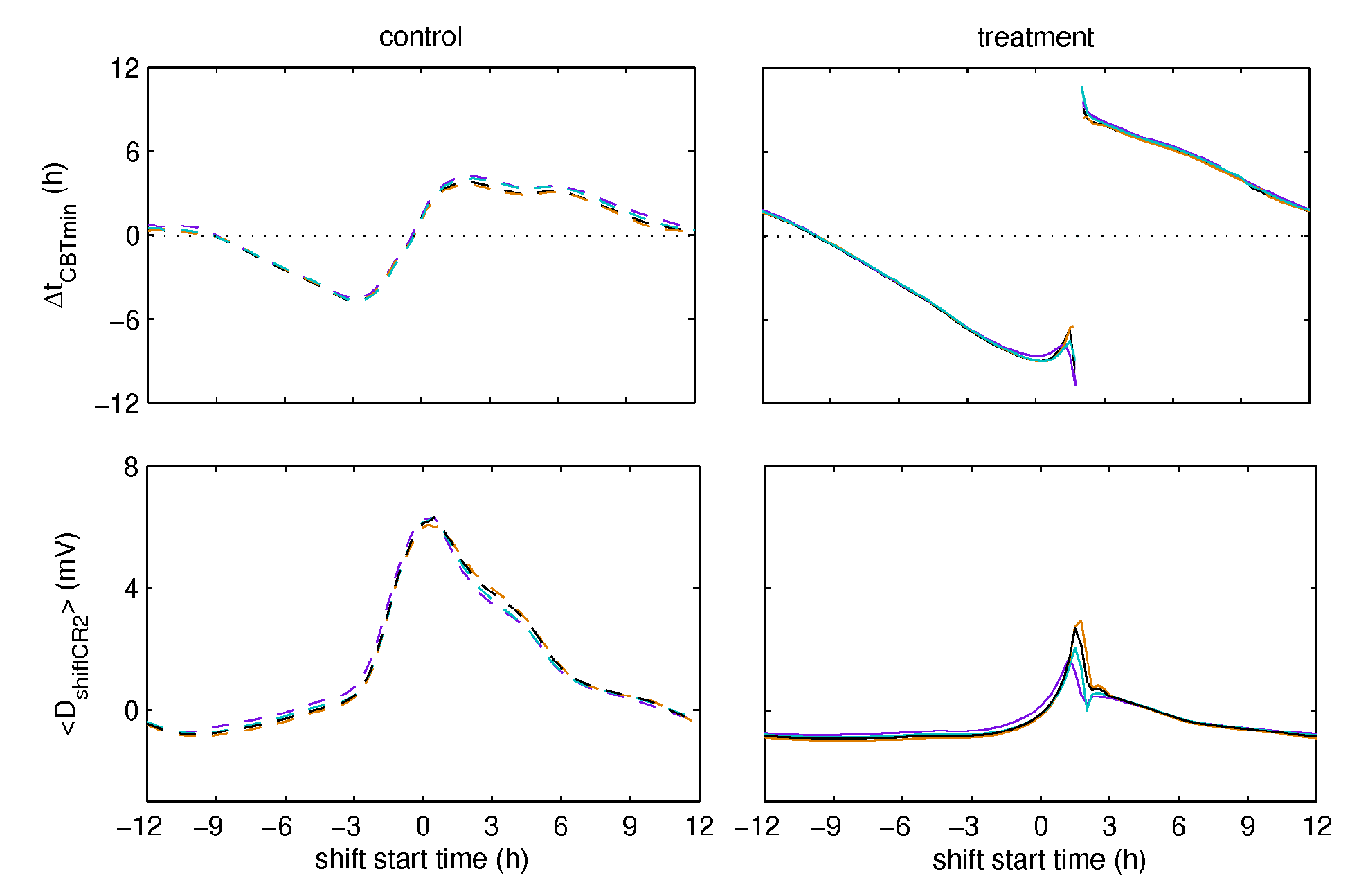

Supplement: Figure S4 — Dependence of adaptation on shift start times for different ambient light profiles and parameter sets according to Table S1. The colors correspond to the different light profiles: red – profile 1, black – profile 2, green – profile 3, blue – profile 4. The black line for light 2 is the same as in Fig. 7 of the paper. Dashed lines refer to the results for control protocol and solid lines to the treatment protocol. (TIF) [file pone.0053379.s004.tif]
