# Supplementary material for: Adaptation to Shift Work: Physiologically Based Modeling of the Effects of Lighting and Shifts’ Start Time
Source: PLoS One. 2013 Jan 4;8(1):e53379. doi: 10.1371/journal.pone.0053379 (PMC3537665; doi:10.1371/journal.pone.0053379)
Supplement: Table S1 — Parameter values corresponding to the ambient light profiles shown in Fig. S3. The parameter sets are chosen in accord with conditions outlined in “Parameter adjustment” section. Profile 2 and the corresponding parameter set (set A in Fig. S1) are the ones that are used throughout the paper. (DOC) [file pone.0053379.s005.doc]

|  | **profile 1** | **profile 2** | **profile 3** | **profile 4** |
| --- | --- | --- | --- | --- |
| *χ* | 44 | ***51*** | 57 | 48 |
| *k* | 0.56 | ***0.51*** | 0.495 | 0.522 |
| *q* | 0.56 | ***0.6*** | 0.6 | 0.6 |
