# Supplementary material for: Adaptation to Shift Work: Physiologically Based Modeling of the Effects of Lighting and Shifts’ Start Time
Source: PLoS One. 2013 Jan 4;8(1):e53379. doi: 10.1371/journal.pone.0053379 (PMC3537665; doi:10.1371/journal.pone.0053379)
Supplement: Text S1 — Supporting information on adjustment of the model parameters and model dynamics at different ambient light profiles. (DOC) [file pone.0053379.s006.doc]

Supplementary material for

**Adaptation to shift work: physiologically based modeling of the effects of**

**lighting and shifts’ start time**

Svetlana Postnova^1,2,3,*^, Peter A. Robinson^1,2,3^, Dmitry D. Postnov^4^

^1^School of Physics, The University of Sydney, Sydney, New South Wales, Australia; ^2^Center for Integrated Research and Understanding of Sleep (CIRUS), The University of Sydney, Sydney, New South Wales, Australia; ^3^Brain Dynamics Center, The University of Sydney, Sydney, New South Wales, Australia; ^4^School of Physics, Saratov State University, Saratov, Russia.

* E-mail: [postnova@physics.usyd.edu.au](mailto:postnova@physics.usyd.edu.au)

**Model predictions at different parameter sets**

In order for the model to match the experimental observations it has to satisfy two conditions: (i) sleep at the baseline should be in the range of experimentally observed mean ± SEM, which implies sleep start time between 23:43 and 00:40; (ii) Δ*t_CBT_*_min_ in response to the control protocol should likewise be in the range of the experimentally observed mean ± SEM, which gives 1.01 – 1.19 h. Sleep duration is set to 7.7 h and is not strongly affected by change of *χ, k,* and *q*. Such wide distribution of the allowed dynamics necessarily means that there is a multitude of parameter sets satisfying these conditions. Figure S1 summarizes these parameters sets, which lie in the yellow area.

It has to be noted, that although all these parameter sets satisfy our general conditions for match to the experiment, there are slight variations of the model dynamics at these sets. For example, sleep start time that lead to the shift of the CBT minimum within the required range of 1.01 – 1.19 h are closer to 23:43 at the top border of the yellow area and to 00:40 at the bottom border in Fig. S1. The dashed lines show where the sleep start time is outside the required region, but when the second condition can still be satisfied.

*Dynamics at default parameter values (point B)*

It is immediately apparent that there is no solution for *χ* at the default values of *k*=0.55 and *q=*1/3 (point B in Fig. S1) that satisfies both conditions with the used light profile. At these default values of *k* and *q* the highest value of *χ* that still allows circadian entrainment at the given ambient light exposure is 66.5 hours which leads to baseline sleep onset at 01:12 and Δ*t_CBT_*_min_ =1.9 h. Decrease of *χ* at this *k* and *q* allows achieving the sleep start time that satisfies the first condition, but at the same time lead to further advance of the CBT minimum, meaning that second condition cannot be fulfilled. It is possible, however, that at other ambient light conditions such solutions might be found, but we have not tested this possibility here.

*Dynamics at different parameter sets inside the solutions range*

It can be expected that the predictions in Figs 4-8 of the paper will be similar for different parameter sets, because we did not observe any strong nonlinearities in the system’s response to change of *k, q,* and *χ* in the examined parameters range (see Fig. 9). Nevertheless, we have examined whether predictions hold for different parameter sets by checking the model dynamics at different points of the diagram in Fig. S1. In particular, Figure S2 compares the predictions for the dependence of *Δt_CBT_*_min_ and mean shift sleep drive on the shifts start time at different parameter sets indicated with A, C, D, E and F in Fig. S1. The parameter set A is the same as used throughout the paper. As seen in the figure predictions for adaptation at different shift start times are qualitatively similar for the different parameter sets for both control and treatment protocols.

**Dynamics at different ambient light exposure**

In our study we have tried to reconstruct the experimental protocol in [25] as close as possible by accordingly implementing the data for light intensities and timing of light where provided. However, a number of inputs required for simulation were not recorded in the experiment. One of such inputs is the ambient light exposure during the times when subjects were not in the laboratory. Therefore, in order to see whether our predictions are robust we have examined different ambient light protocols. In particular, we have tested different light intensities and different shapes of light. Some of the examples are demonstrated in Fig. S3.

Since we needed to match the model to experimental data, we have had to slightly adjust the parameters of the model accordingly, such that the baseline sleep and wake onset times, as well as the change of the core body temperature minimum location fall in the range of *mean ± SEM* of the experimental data. Note, that change of the parameters is justified since the subject having the same response to different ambient light condition should have different intrinsic characteristics. Table S1 demonstrates examples of the suitable parameter sets for each of the presented light profiles. Note that for each of these light profiles there is a range of parameters satisfying the experiment, similar to that shown in Fig. S1 calculated for profile 2 in Fig. S3.

In order to check whether the predictions for the model dynamics are robust for these different ambient light profiles we have again examined the dependence of adaptation on the start time of the shifts. The model predictions for these different light profiles are compared in Fig. S4. As can be seen from the figure, as soon as the parameters of the model are adjusted in accord with experimental observations, the other predictions of our model remain very nearly the same for different ambient light conditions. The plots overlap so much that it is even hard to distinguish them.

Obviously there is an unlimited number of possible ambient light profiles, and we cannot test them all. In all the light profiles that we have examined the maximum light intensity is higher than lighting during the shifts in control protocol, and “sunrise”/“sunset” times are always the same. However, given that (i) different parameter sets satisfying the match to experiment at a given light profile lead to the same predictions for optimal and worst shift times (Fig. S2), and that (ii) at different ambient lighting the model leads to the same predictions once the parameters are adjusted (Fig. S4), it is possible that the predictions will also hold for other ambient light conditions as long as the ambient lighting is the same during baseline and shifts protocol.
